# The Arthroscopic Intra-Articular Stabilization of the Shoulder for Irreparable Rotator Cuff Tear: A New Technique Proposal

**DOI:** 10.3389/fsurg.2021.624100

**Published:** 2021-12-06

**Authors:** Pierfrancesco Luciani, Luca Farinelli, Luca De Berardinis, Antonio Gigante

**Affiliations:** Clinical Orthopaedics, Department of Clinical and Molecular Sciences, UniversitàPolitecnicadelle Marche, Ancona, Italy

**Keywords:** rotator cuff tear, technique, arthroscopic stabilization, neoligament, shoulder

## Abstract

**Background:** Superior capsular reconstruction (SCR) has shown good results in the management of irreparable rotator cuff tears due to the depressive effect on the humeral head, but it is a technically demanding and expensive procedure.

**Purpose:** We hypothesized that an intra-articular neoligament that prevents the superior translation of the humeral head could give similar results in terms of the superior translation of humerus (STH) and range of motion (ROM).

**Study Design:** To compare our proposed technique and the SCR, we conducted a biomechanical study on 10 porcine shoulders in a custom shoulder testing system.

**Methods:** STH and total rotational ROM were quantified in the following four scenarios: (1) when the rotator cuff was intact, (2) after cutting the supraspinatus tendon, (3) after the reconstruction of the superior capsule by long head of the biceps tendon (LHB), and (4) after an arthroscopic intra-articular stabilization by an intra-articular graft. Our proposed technique provides the creation of a humeral and glenoid tunnel, the passage of a graft through these tunnels under arthroscopic guidance, and the graft fixation in the two tunnels. We analyzed the STH and total ROM in each scenario.

**Results:** With respect to the STH, we reported that the present proposed technique is characterized by a significant reduction of superior translation at 0 and 45° compared to scenario 2. In addition, the comparison between our proposed technique and SCR showed a significant difference of the STH at 0° of abduction. Total rotational ROMs of the two tenchinques were similar to scenario 2. Therefore, the use of an intra-articular ligament that prevents the STH can restore shoulder stability in irreparable rotator cuff injuries at both 0 and 45° of glenohumeral abduction without apparently limiting the total rotational ROM.

**Conclusion:** Our proposed technique could be an important treatment option in irreparable rotator cuff tears, especially in patients under 65 years in whom reverse shoulder arthroplasty (RSA) has shown poor results and many complications.

## Introduction

Massive rotator cuff tears could determine significant pain and disability of the shoulder (1). These lesions are often characterized by chronic inelastic retraction or significant muscle fat degeneration that can preclude successful repair (2–7). Treatment options for massive and irreparable rotator cuff injuries depend on a multitude of factors including age of the patient, activity level and expectation, degree of joint arthropathy, and the extent of disability caused by the injury (8). In these lesions, surgical options include arthroscopic debridement, biceps tenotomy/tenodesis, partial cuff repair, interposition grafts, subacromial balloon spacers, tendon transfers, superior capsular reconstruction (SCR), and reverse shoulder arthroplasty (RSA) (9, 10). Although RSA has shown excellent results in irreparable rotator cuff tears in patients over 65 years, in younger patients, it has not been as successful with complication rates up to 50% (11–13) and with unsatisfactory clinical outcomes (14). The superior translation of the humeral head following massive rotator cuff tears causes impaired glenohumeral joint mechanics, shoulder dysfunction, and, in the long term, the development of glenohumeral arthrosis. Studies on SCR have shown that returning the humeral head to its original position restores joint biomechanics and improves clinical outcomes (15, 16).

Nowadays, SCR is to be considered a technically demanding and onerous procedure due to the cost of the allograft and the devices used to fix it and the prolonged operating time (17).

Therefore, recent biomechanical studies have used the long head of the biceps tendon (LHB) for the superior capsular reconstruction showing the stability of the glenohumeral joint (18–21).

In this study, given the excellent clinical outcomes obtained by the SCR due to the depressant effect on the humeral head, we hypothesized that similar results can be obtained using an intra-articular graft that prevents the superior translation of the humeral head and at the same time, it is able to balance the pairs of forces of the anterior and posterior cuff. Therefore, we proposed an arthroscopic intra-articular stabilization of the shoulder named as the AISS technique. The aim of this biomechanical study was to compare the STH and total range of motion (ROM) of porcine shoulder in case of irreparable rotator cuff tears treated by our proposed AISS technique and the SCR technique with LHB tendon described by Chillemi et al. (17) known as the Arthroscopic Biceps Chillemi (ABC) technique.

## Materials and Methods

### Specimen Preparation

**Figure 1:**
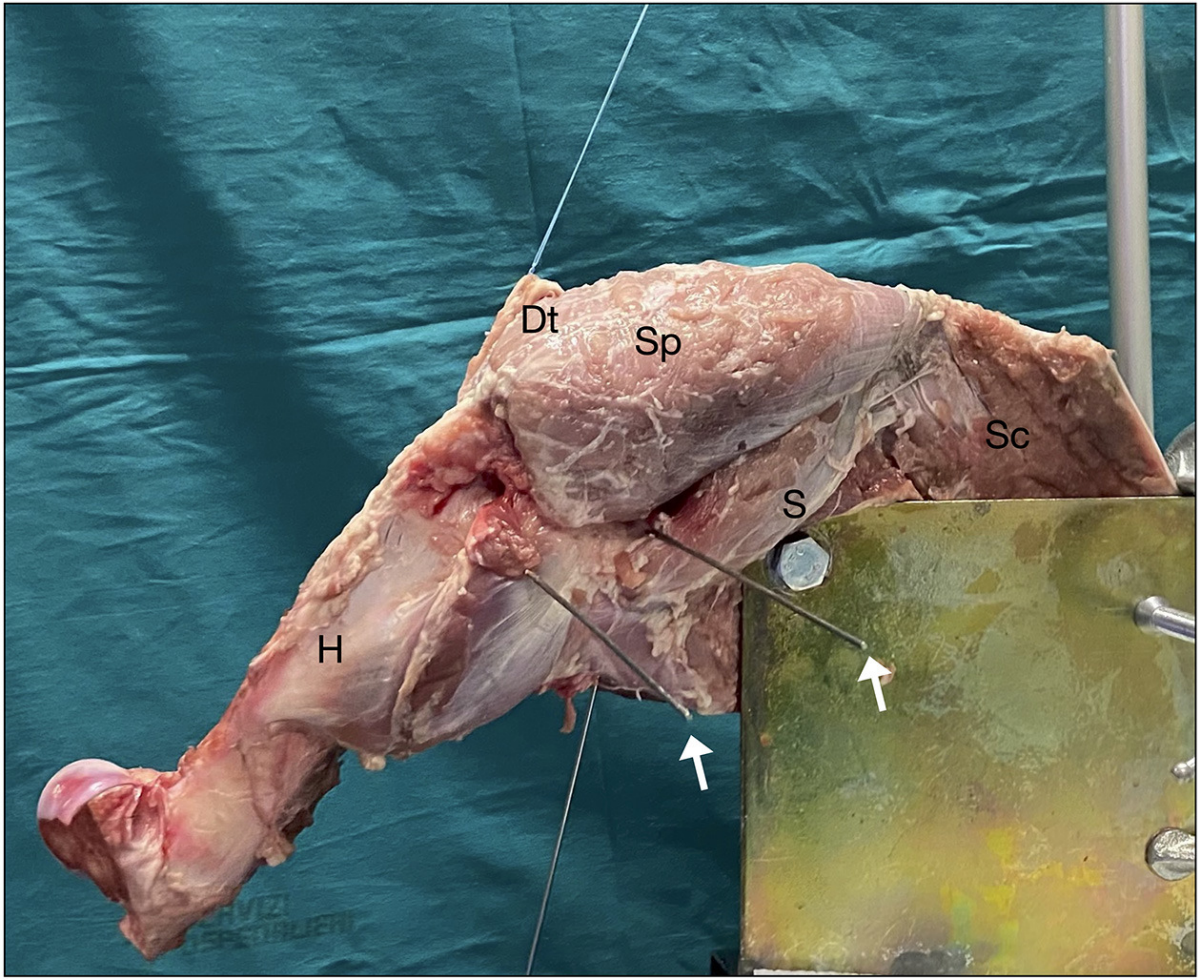
Shoulder fixed to the system prepared for test. The deltoid tendon (Dt) is basted for the application of forces. White arrows: K-wires located as explained in the text. Sp, sopraspinatus; S, subscapularis; SC, scapula; H, humerus.

This study was conducted on 10 frozen adult porcine shoulders. Specimens were thawed at room temperature on the night before dissection and testing. The skin, subcutis, and muscles were removed from each sample, while the supraspinatus, teres minor, infraspinatus, subscapularis, teres major, coracobrachialis muscles, and the deltoid distal tendon insertion were preserved. A No. 2 FiberWire suture (Arthrex Incorporation, Naples, Florida) was used to tie a krackow locking-running stitch to the tendinous insertion for muscle loading during testing. The scapula was fixed to a custom metal plate by using three large bolts (Figure 1). The scapular plate was fixed to a custom testing jig in 20° of anterior tilt in the sagittal plane. A goniometer was placed at the distal end of the humerus to measure humeral rotation. A torque dynamometric wrench was used to apply a predetermined torque for measuring rotational ROM. Muscle forces were simulated via a fishing line attached to No. 2 FiberWire suture, which was attached directly to the tendon. Adjustable pulleys and a positioning plate were used to approximate physiological muscle force vectors without friction. The authors PL and AG performed all the tests.

### Testing Conditions

**Figure 2:**
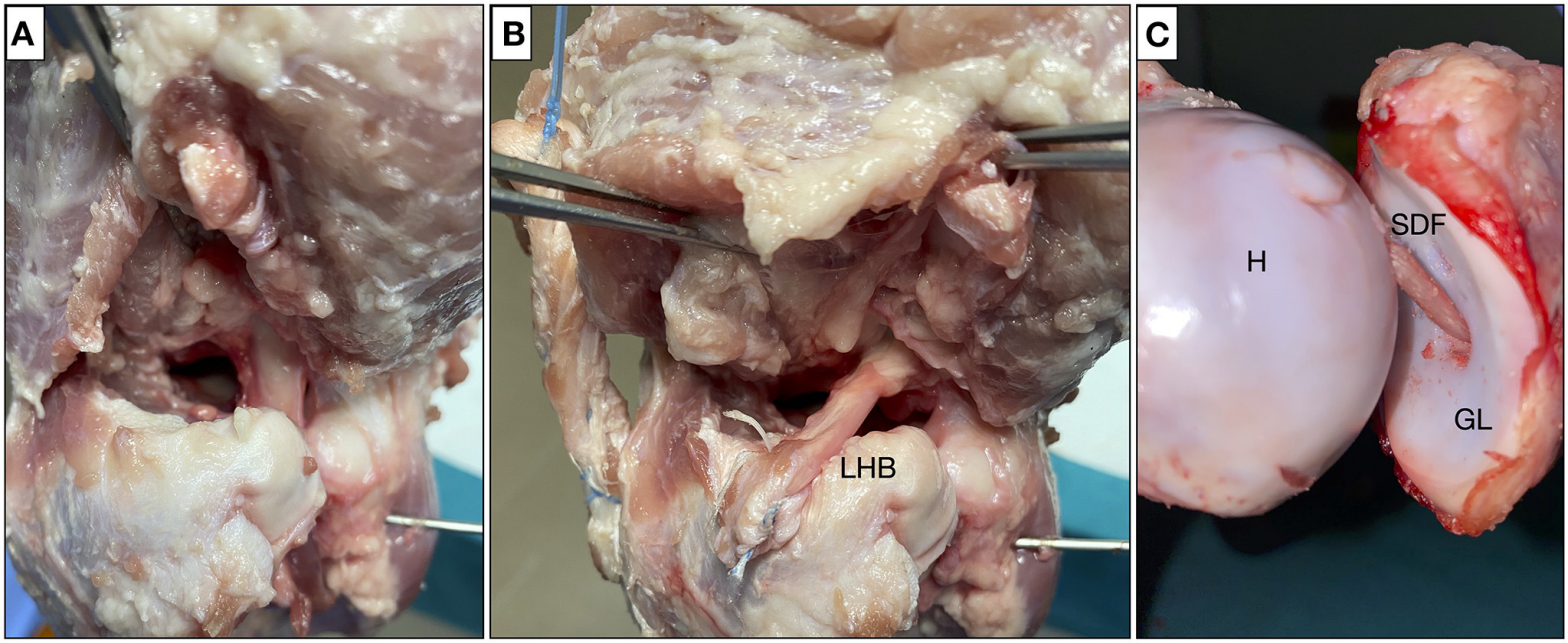
Testing scenario: **(A)** complete tear of the supraspinatus; **(B)** SCR with ABC technique; and **(C)** stabilization with the AISS technique. SCR, superior capsular reconstruction; ABC, Arthroscopic Biceps Chillemi; AISS, arthroscopic intra-articular stabilization of the shoulder; LHB, long head biceps tendon; SDF, superficial digital flexor tendon; H, humerus; GL, glena.

Every biomechanical test is performed in each of the following four scenarios:

Scenarion 1: The rotator cuff is intact as control.
Scenarion 2: An irreparable supraspinatus tear model has been made cutting the supraspinatus and superior capsule at the insertion on the greater tuberosity to the glenoid along the anterior and posterior edges of the supraspinatus tendon. To simulate an irreparable rotator cuff tear, the lateral edges of the supraspinatus and superior capsule were retracted to the glenoid (Figure 2A).
Scenarion 3: A SCR was performed by using a LHB tendon as described in the ABC technique (17) (Figure 2B). The graft length was determined to be 15 mm longer than the length from the superior glenoid to the lateral edge of the greater tuberosity at 30° of glenohumeral abduction. The proximal insertion of the tendon over the glena was left intact, while on the greater tuberosity the tendon was attached with two anchors (ReelX STT 4.5 mm, Stryker, Kalamazoo, MI). The graft was attached at 30° of glenohumeral abduction in the scapular plane (equivalent to 45° of shoulder abduction), as described in the original technique.
Scenarion 4: LHB tendon was removed and intra-articular shoulder arthroscopic stabilization is performed (AISS technique) (Figure 2C).

### Surgical Technique

**Figure 3:**
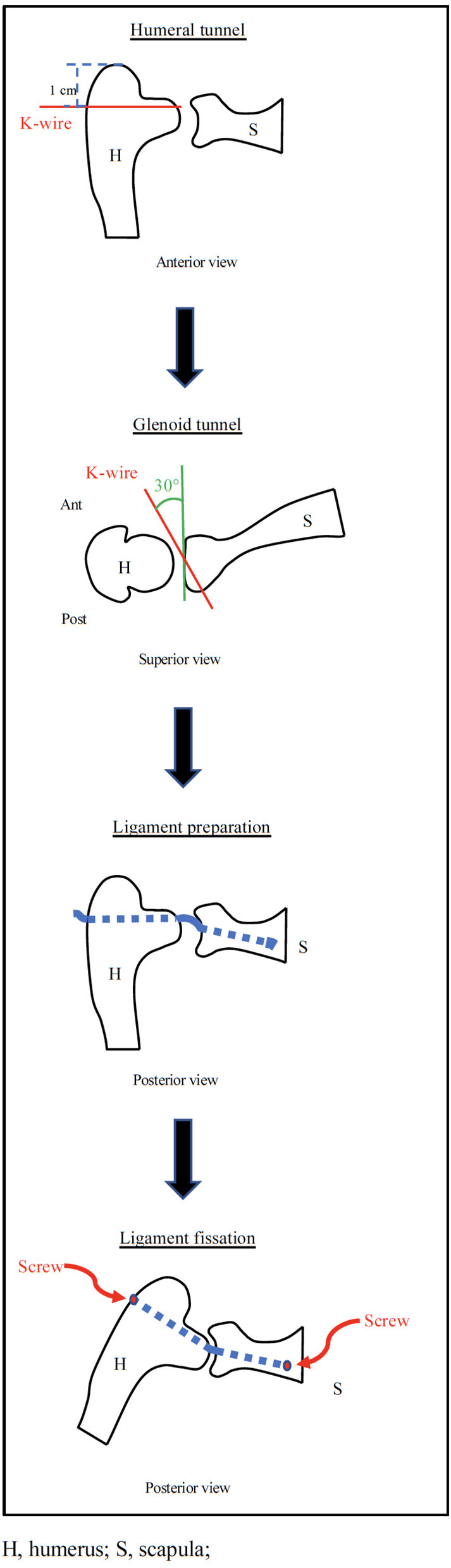
Diagramatic representation of the AISS technique.

A proposed surgical technique steps are given in Figure 3. The steps are explained as following.

#### Humeral Tunnel

**Figure 4:**
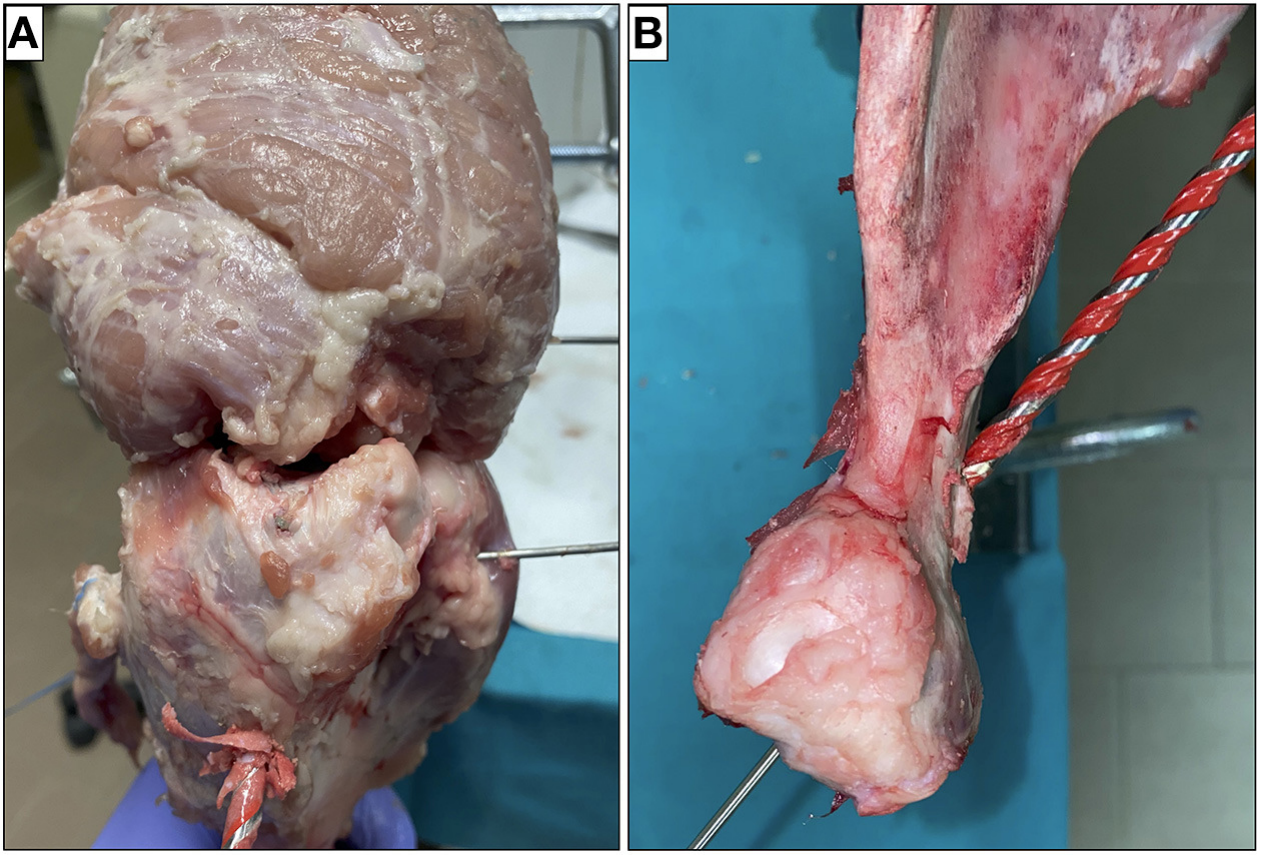
Humerus **(A)** and glena **(B)** were drilled on the K-wire guide.

A K-wire was inserted under fluoroscopic control 1 cm distal to the superior margin of the greater tuberosity with humerus positioned at 0° of abduction and neutral rotation. The wire must be parallel to the ground, follow the humeral retroversion of 20°, and protrudes on the anatomical surface of the humerus at the height of the superior margin of the glena. At this point, a 5-mm hole was drilled on the wire guide. The length of the tunnel is approximately 8.5 cm in porcine shoulder (Figure 4A).

#### Glenoid Tunnel

After creating a standard posterior arthroscopic portal (for optics), an anterosuperior arthroscopic portal was performed. Through this portal, a K-wire was pointed at the glenoid fovea. The K-wire was inserted into the glena maintaining an inclination of 30° with respect to the sagittal plane and 10° to the axial plane in a posteroinferior direction. The wire will come out at the level of the scapula neck between the lower edge of the neck and the spine of the scapula.

At this point, we proceeded to execute a posterior miniaccess centered on the K-wire. The suprascapular nerve was identified and carefully isolated and a 5-mm hole was drilled on the wire guide. The length of the tunnel is about 3 cm (Figure 4B).

#### Ligament Preparation

**Figure 5:**
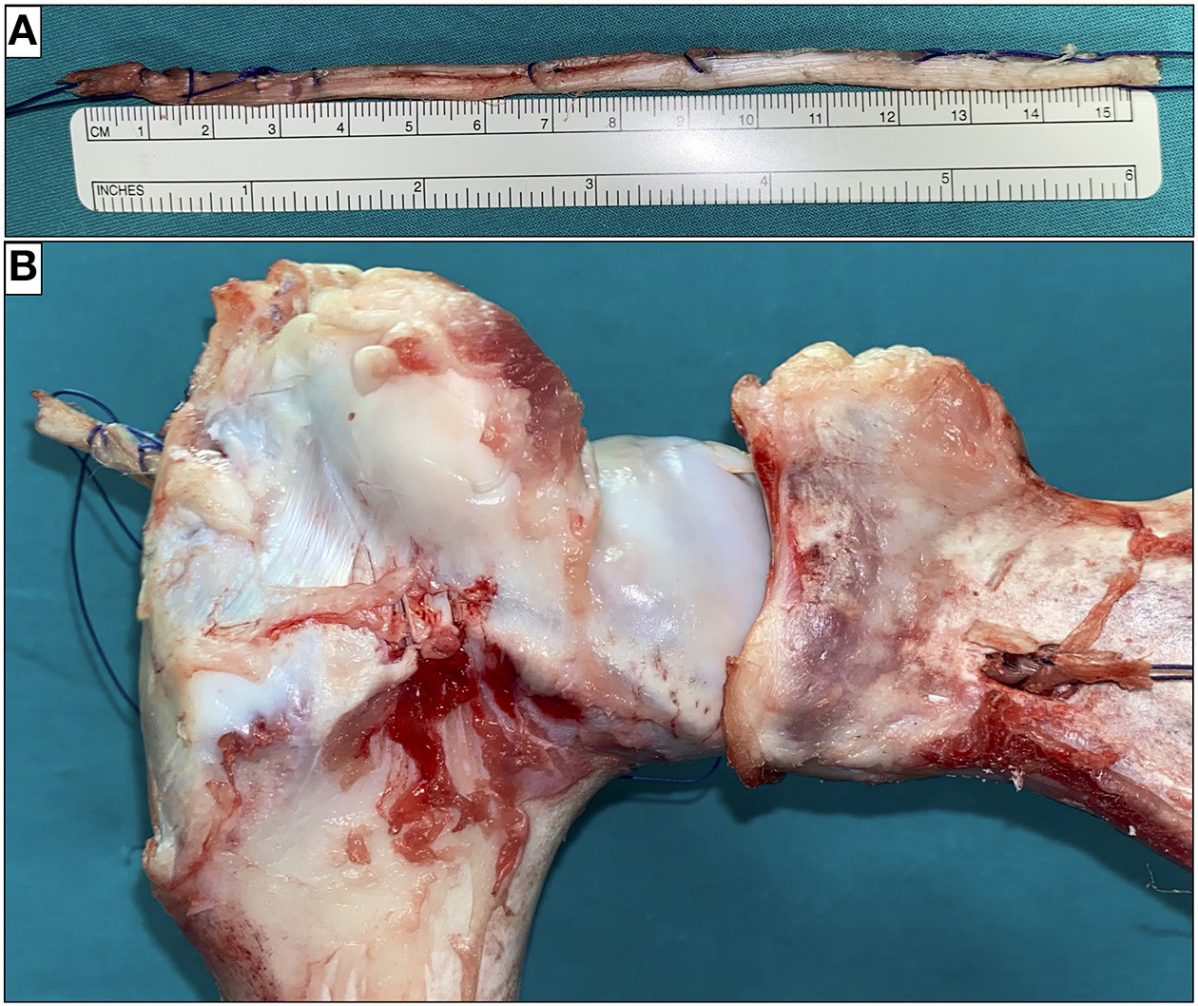
The ligament was basted with absorbable wires **(A)** and passed through the two tunnels **(B)**.

A ligament of about 15 cm by 5 mm in diameter (obtained from the porcine superficial digital flexor tendon) was basted with absorbable wires (Figure 5A) and was passed before in the humeral tunnel and then in the glenoid tunnel under arthroscopic vision (Figure 5B).

#### Ligament Fixation

**Figure 6:**
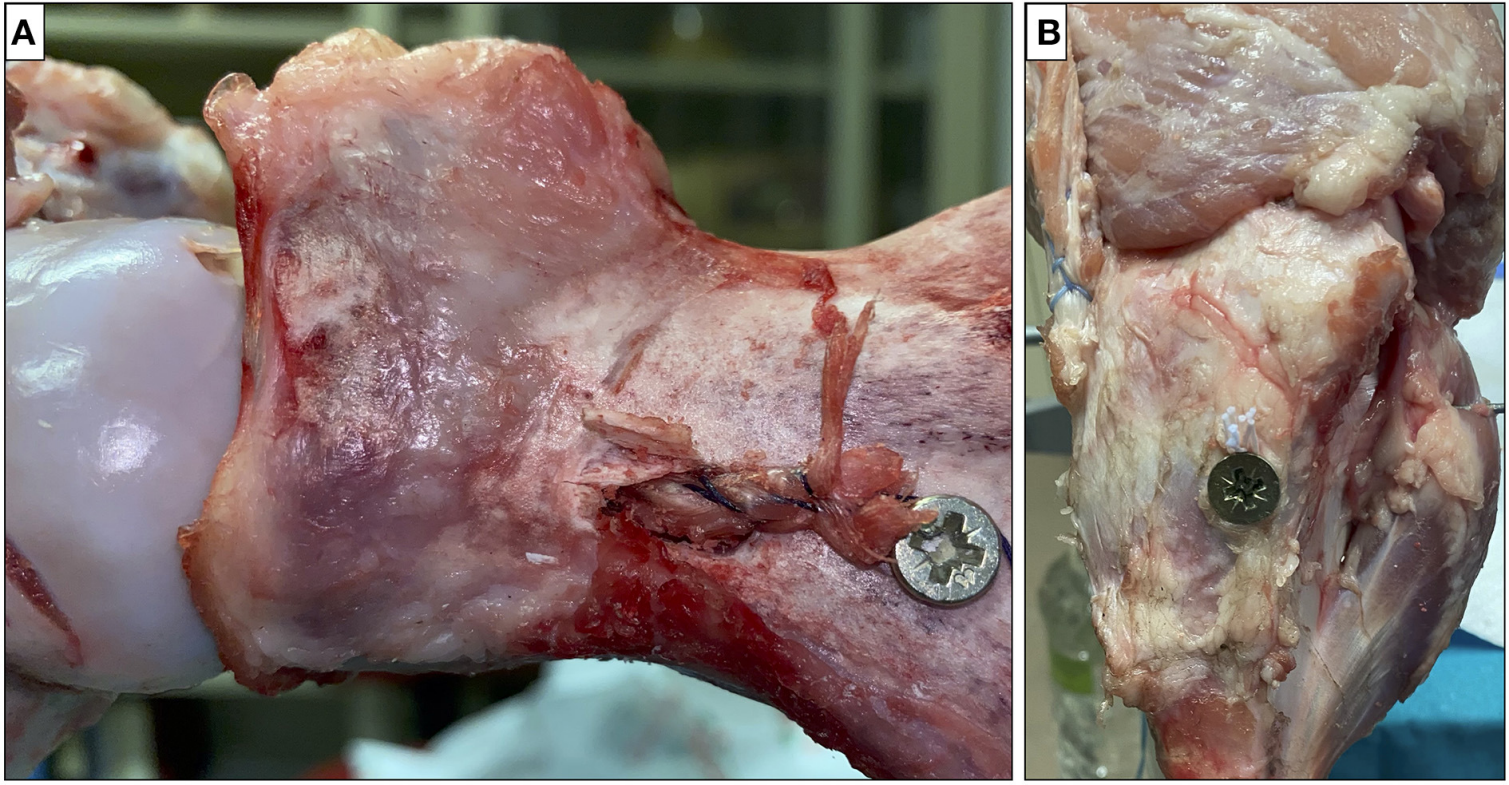
The ligament was fixed at the scapula **(A)** and humerus **(B)** with screws.

Many devices could be used for ligament fixation to the glena as a staple or a screw. We have excluded indirect fixations such as Endobutton or TightRope given the shortness of bone tunnel and direct fixations to the glenoid fovea in order not to preclude the future possibility of a reverse prosthesis. In this study, the ligament was fixed to the scapula using a 4-mm screw inserted from a posterior miniaccess taking care not to damage the suprascapular nerve (Figure 6A).

Finally, the ligament was tensioned and fixed to the humerus with a 4-mm interference screw at 30° of glenohumeral abduction (about 45° of shoulder abduction) (Figure 6B) as suggested by SCR technique (22).

### Measurements

All the measurements were performed at 0 and 45° of glenohumeral abduction and with neutral humeral rotation. For each position, it has been used for two different loading conditions: in condition n.1 (named rest condition), the deltoid was subjected to a force of 40 N, while in condition n.2 (named stress condition), the deltoid was subjected to a force of 80 N because we would simulate the superior translation force in order to evaluate the stability of the shoulder.

The amount and direction of translation relative to the initial position were quantified using K-wires placed on the midpoint of the anterior glenoid region and in the proximal portion of the bicipital groove by a three-dimensional digitizer (Micro-Scribe 3DLX; Immersion Corporation, San Jose, USA); the accuracy of this device was 0.30 mm according to the manufacturer. The location of the humeral head relative to the glena was recorded under loading conditions N.1 and N.2. To evaluate superior shoulder stability, superior translation of the humerus was calculated by comparing the distance between two wires in the superior-inferior direction under loading condition 1 with that under loading condition 2 in all the positions and cases previously described.

Total rotational ROM was calculated by adding external and internal rotational ROMs using a 360° digital goniometer (GemRed). Measurements were performed only in the loading condition 1 because when the head of the humerus has migrated superiorly, making extreme movements that could cause ruptures of the ligament, with consequent inconsistent data.

### Statistical Analysis

All the measurements were repeated three times. The data were collected and organized with Excel (Microsoft, Redmond, Washington, USA). Continuous variables were expressed as mean ± SD. The superior translation of the humerus and the rotational ROM in the four conditions were compared with the Mann–Whitney *U* test. Statistical analysis were performed using the SPSS (version 21.0; IBM Corporation, Armonk, New York, USA). *p* < 0.05 was considered as statistically significant.

## Results

### Superior Translation of the Humerus

The creation of a complete and irreparable lesion of the supraspinatus increases the superior translation of the humerus significantly by 2.5 mm at 0° of abduction (*p* < 0.001) and by 2.6 mm at 45° of abduction (*p* < 0.001). Both the ABC and the AISS technique make the shoulder more stable compared to scenario 2. No statistically significant differences have been reported compared to the intact cuff. Additionally, it has been observed a reduction of the glenohumeral superior translation of 0.6 mm with the AISS technique compared to the ABC one at 45° of abduction (*p* > 0.07). This difference reaches 0.8 mm at 0° of abduction (*p* < 0.04) (Table 1).

**Table 1:** Superior translation of the humerus (STH).

	**Intact**		**Supraspinatus tear**		**ABC technique**		**AISS technique**	
	**STH,** **mm**	**STH,** **%**	**STH,** **mm**	**STH,** **%**	**STH,** **mm**	**STH,** **%**	**STH,** **mm**	**STH,** **%**
0° abduction	1.8 ± 0.7	100	4.3 ± 0.7^a^	238	2.5 ± 0.4^b^	139	1.7 ± 0.4^b^^,^^c^	94
45° abduction	2.10 ± 6	100	4.70 ± 7^a^	223	2.60 ± 3^b^	123	20 ± 5^b^	95

*Values are expressed as mean ± SD. “STH, %” was calculated by dividing each value by that for the intact condition at the same position*.

a *Statistically significant difference compared with intact condition (p < 0.05)*.

b *Statistically significant difference compared with simulated supraspinatus tear (p < 0.05)*.

c *Statistically significant difference compared with the Arthroscopic Biceps Chillemi (ABC) repair (p < 0.05)*.

### Total Rotational ROM

In scenario N.2, the total rotational ROM of the shoulder increases by 16 and 19°, respectively, at 0° (*p* < 0.01) and 45° (*p* < 0.006) of abduction compared to healthy rotator cuff. In scenario N.3, there is a nonsignificant decrease in rotational ROM compared to scenario 2 at 0 and 45° of abduction. Similarly, in scenario 4, it has been observed a decrease of rotational ROM of shoulder of 2 and 3°, respectively, at 0 (*p* > 0.46) and 45° (*p* > 0.35) of abduction.

Summarizing, both in the scenario N.3 and N.4, the rotational ROM is significantly greater than that of intact cuff, increasing by 12 and 15° at 0 (*p* < 0.03) and 45° (*p* < 0.01) of abduction with the ABC technique and 14 and 16° at 0 (*p* < 0.02) and 45° (*p* < 0.01) of abduction with the AISS technique (Table 2).

**Table 2:** Total rotational range of motion (in degrees).

	**Intact**	**SupraspinatusTear**	**ABC technique**	**AISStechnique**
0° abduction	10 ± 6	26 ± 5^a^	22 ± 4^a^	24 ± 4^a^
45° abduction	24 ± 7	43 ± 5^a^	39 ± 4^a^	40 ± 5^a^

*Values are expressed as mean ± SD*.

a *Statistically significant difference compared with intact condition (p < 0.05)*.

## Discussion

The most important finding of this study was that the use of an intra-articular graft that prevents superior translation of the humerus could restore shoulder stability in irreparable rotator cuff injuries at both the 0 and 45° of glenohumeral abduction without apparently limiting the total rotational ROM in porcine model. In this biomechanical study, the AISS technique and the intact cuff gave similar results on superior humeral translation. The use of the LHB tendon for the SCR has considerably reduced humeral elevation, but it has not restored the condition given by the intact cuff. This residual superior instability could lead to abrasion and rupture of the LHB tendon after surgery with a higher revision rate than the AISS technique. To date, fascia lata and human dermal graft could be used in SCR (23–26). However, concerns about cost, donor site morbidity in case of autograft, increased surgical time and the significantly elongation of dermal graft during test need to be considered (27). Furthermore, an unstable shoulder works with an altered biomechanics and will undergo arthritic degeneration (15, 16, 19).

Comparing the two repair techniques, the AISS gave better results on superior humeral translation than the ABC technique, but this finding was significant only at 0° of abduction. Previous biomechanical studies on the role of the rotator cuff tendon in shoulder elevation have shown that the rotator cuff tendon is more important in initiation of elevation than at a higher angle of abduction (28). Therefore, restoration of glenohumeral joint stability after the AISS at a lower angle of abduction may occur because the neoligament supports the function of a partially repaired rotator cuff tendon as a superior stabilizer at initiation of shoulder elevation.

Excessive traction of the graft could cause stiff shoulders after surgery. When a complete lesion of the supraspinatus tendon is created, there is a significant increase in the total rotational ROM of the humerus compared to the intact cuff (29). In this study, this parameter in both the techniques remains similar to that of the injured cuff. This suggests that both the interventions should not cause stiffness. Clearly, the porcine shoulder has a lower rotational ROM than humans (141° in humans vs. 10° in porcine at 0° abduction) and, therefore, this finding must be confirmed with human studies by plain radiograph and analysis of ROM (19).

The strength of this biomechanical study is the direct measurement of the superior humeral translation and of the rotational ROM, which is not possible in the living. Moreover, this study is consistent because the exact same force can be applied in the same direction for all the specimens and all the four scenarios were tested in each shoulder.

This study had some limitations. Firstly, it was conducted on porcine shoulders, which have anatomical and biomechanical differences from human ones, but porcine models have been commonly used in biomechanical studies for the initial evaluation of rotator cuff reconstruction techniques (30). Muscle loading was static rather than dynamic and this experiment has been performed only in two muscle loading conditions. We did not compare the AISS technique with SCR using fascia lata and human dermal graft because concerns about cost and the significantly elongation over time limit their use.

In addition, the effect of biological healing cannot be evaluated. Lastly, the execution of the humeral tunnel may result in articular cartilage damage in humeral side that could contribute to cartilage degeneration and glenohumeral arthritis. However, it should be pointed out that in massive rotator cuff tears, the cartilage damage is already present. Moreover, the tunnel is small sized and located in the superior part of humeral head.

## Conclusion

Our proposed technique could be an important treatment option in irreparable rotator cuff tears, especially in patients under 65 years in whom RSA has shown poor results and many complications. Obviously, further biomechanical studies on human cadaver are mandatory in order to assess the feasibility, safety, and effectiveness of the AISS technique on massive rotator cuff tears.

## Data Availability Statement

The original contributions presented in the study are included in the article/supplementary material, further inquiries can be directed to the corresponding author/s.

## Ethics Statement

Ethical review and approval was not required for the animal study because the study was conducted on porcine shoulder provided by Butcher's shop.

## Author Contributions

AG had the idea, intuition, and supervised. LF, PL, and LD elaborated the project and made the experiment. PL and LD wrote the manuscript. LF corrected the paper. All authors contributed to the article and approved the submitted version.

## Conflict of Interest

The authors declare that the research was conducted in the absence of any commercial or financial relationships that could be construed as a potential conflict of interest.

## Publisher's Note

All claims expressed in this article are solely those of the authors and do not necessarily represent those of their affiliated organizations, or those of the publisher, the editors and the reviewers. Any product that may be evaluated in this article, or claim that may be made by its manufacturer, is not guaranteed or endorsed by the publisher.
